# Outpatient dental care for people with disabilities under general anaesthesia in Switzerland

**DOI:** 10.1186/s12903-020-01203-6

**Published:** 2020-08-18

**Authors:** Julia Jockusch, Bernhard A. J. Sobotta, Ina Nitschke

**Affiliations:** 1 Clinic of General, Special Care and Geriatric Dentistry, Center of Dental Medicine, University of Zurich, Plattenstrasse 11, CH-8032 Zurich, Switzerland; 2 Department of Prosthodontics and Materials Science, Gerodontology Section, University of Leipzig, Liebigstraße 12, 04103 Leipzig, Germany

**Keywords:** Anaesthesia/sedation, Dental treatment, Hospital dentistry, People with disabilities/disabilities

## Abstract

**Background:**

Life expectancy of people with permanent disabilities has increased. The dental care of these vulnerable patients is one of the greatest challenges for the dentist and the dental team due to limited or non-existent cooperation and the associated lack of health competence. In order to be able to provide safe and acceptable, quality dental treatment without psychological and physical stress for these patients, it is therefore necessary to resort to sedation or general anaesthesia (GA) under medical supervision. The aim of the analysis is to highlight the need for dental treatment performed under GA for people with disabilities and the associated indications and treatment patterns.

**Methods:**

Ten-year retrospective analysis of outpatient dental care under GA for people with disabilities.

**Results:**

Of all adult patients (*n* = 221) who attended the GA pre-assessment, 69.7% (*n* = 154) received dental treatment under GA based on the clinical findings or in cases of suspected pain. Most patients received one GA. A total of 205 dental treatment sessions were performed under GA mostly for conservative (*n* = 442, 52%) and surgical (*n* = 389, 45.8%) procedures. Endodontic treatment (*n* = 19, 2.2%) was rare. The failure rate related to all teeth in need of treatment (*n* = 850) was 5.1% (*n* = 43), in most cases due to secondary caries (*n* = 40; 93.0%). Patients were enrolled in an annual recall for dental examination and prophylaxis without GA. Non-compliant patients for whom oral hygiene was impossible received a periodic GA.

**Conclusion:**

There is a high need of people with disabilities for dental treatment under GA. Main indications for treatment under GA are dental complaints, pain or suspected pain. Dental care can be successful if, for the benefit of patients with special needs, all carers cooperate closely. Caregivers have to be trained in nutrition control as well as in oral hygiene. These factors in conjunction help to prevent dental emergencies.

## Background

The life expectancy of people with permanent disabilities has increased due to good medical and social care. These vulnerable patient groups frequently face barriers to oral healthcare (e.g. difficulties accessing care, lack of availability of appropriate care) [1, 2]. The dental care of these patients poses a challenge for the dentist and the dental team [3] due to limited or non-existent cooperation of patients and the associated lack of health competence. In many cases, the disabilities also result in a reduced ability to maintain oral hygiene, either by the patient him- or herself or through third parties [4]. This often leads to oral diseases (caries, periodontitis, etc. [5–7]), which increase the need for dental treatment [5, 8, 9]. In addition, people with intellectual disabilities are often unable to recognise dental problems and/or oral pain or discomfort. Effective communication may not be achievable for both the dentist and the caregivers/legal guardian for people with disabilities. This often makes a participative therapy decision involving the patient impossible. Furthermore, diagnostic measures (e.g. intra-oral examination, x-rays) may be difficult or even impossible to perform. Even for experienced specialists and dental teams, chairside treatment in the usual setting is often not feasible due to a lack of patient participation [10]. In order to be able to provide high-quality, safe and acceptable dental treatment [3] without psychological and physical stress for these patients, it is therefore necessary to resort to sedation or general anaesthesia (GA) [11–14]. The dentist has to decide for or against dental treatment under GA without - in most cases – having an exact intra-oral examination as a basis for this decision [15, 16]. The primary indication for dental treatment under GA in the literature is the lack or absence of patient cooperation [4, 9, 11, 17–20].

A large number of studies investigated the outcomes of dental treatment under GA in children [21–24]. However, little information is available on the treatment of people with disabilities under GA [25, 26] in spite of the known benefits of dental treatment in GA which can improve patient safety and therapy outcome (exact planning, lege artis treatment, etc.) [27].

Under GA several oral problems can be treated within only one session. This is associated with a reduction of stress for the patient and the accompanying person as well as of costs and transport requirements [16]. Treatment under GA enables the operator to provide high quality restorative care and thus allows treatment outcomes that are comparable with those of persons without disabilities during chairside dental treatment. The ethical principle of justice [28], which in this context demands that all people, with and without disabilities are treated equally with the same methods and treatment options, makes the use of GA essential for many cases. People with disabilities should receive the same quality and outcomes in treatments under GA [29, 30] as people without disabilities with chairside treatment.

The medical elimination of pain (analgesia) with simultaneous sedation or attenuation of consciousness (analgosedation) needs to be discussed as an alternative for treatment under GA. Especially oral sedation, which can be prescribed in Switzerland only by a medical doctor and not by the dentist, may be advantageous due to the ease of application. Oral sedation does not require additional personnel and material resources, which makes it a cost effective option. However, oral sedation may be difficult to control and adjust to patients’ individual needs. Other methods of sedation which can be used by a trained dentist, e.g. titratable methods such as nitrous oxide, are characterized by a precise control of the duration and depth of the sedation. Nevertheless, its use in people with disabilities, especially in people with intellectual disabilities, is limited as they frequently find it difficult to tolerate the mask. Manley et al. describe a technique of sedation using Midazolam administered orally or intranasally which provides sufficient sedation before an intravenous sedation is administered. This approach may be more appropriate for use in people with challenging behaviour [31].

When sedation is possible, it can improve patient compliance and reduce stress levels and therefore allow chairside treatment. Depending on the sedation method used it might be advisable or legally required in individual countries to have medical supervision present during sedation as is the case with intravenous sedation in Switzerland.

GA is the alternative for all other cases. Possible GA risks and complications should not be underestimated, especially in vulnerable patient groups. Cavaliere et al. reported increased blood pressure and heart rates associated with a statistically significant increase of cortisol and prolactin levels [32]. Even though such events in people with intellectual disabilities, trisomy 21 or dementia may not pose a risk in the absence of other medical conditions, patients with physical limitations need special attention [33]. Studies have demonstrated an increased risk of morbidity with increasing depth of anaesthesia. However, it is not clear whether sedation in special needs patients has a general advantage over GA [33]. A risk of sedation is the lack of airway safety when reflexes are reduced or no longer present. The final decision for GA or sedation in Switzerland is made by the anesthetist who largely bases his decision on the medical risks associated with GA. The authors favour GA treatment, especially for vulnerable patients such as people with disabilities, if medically justifiable.

The aim of the presentation is to highlight the need for dental treatment under GA for people with disabilities and the associated indications and treatment patterns.

## Material and methods

A 10-year (2007–2017) retrospective analysis of dental records of adult people (≥ 18 years) with disabilities who underwent outpatient GA for dental treatment was made at a specialised clinic in Switzerland. All records of adult patients who underwent a GA pre-assessment were included.

All data were extracted manually from the files using a standardised form. In addition to sociodemographic data (age, sex, oral functional capacity [34], etc.), data on dental treatment under GA (subjective reasons for treatment provided by relatives or carers and objective indication provided by the dentist as documented on file, waiting time between initial GA pre-assessment and first treatment under GA, DMF/T index, procedures performed, failures, etc.) were recorded. Disability was classified by judgement of the authors based on the availability of the patients` medical diagnoses in eight groups - congenital brain injury, epilepsy, acquired disability, intellectual disabilities not further specified, syndromes, autism, trisomy 21, others.

Oral functional capacity (OFC) (Table 1) [34] was used to assess patients from the multifactorial perspective of a gerostomatological dentist working in special care. OFC is an assessment tool which helps to assess patients with regard to their resilience capacity level (RCL) with the three parameters therapeutic capability, oral hygiene ability and self-responsibility. The levels of the parameters therapeutic capability and oral hygiene ability range from 1 - normal to 2 - slightly reduced, 3 - greatly reduced and 4 - none. Self-responsibility is recorded with the capacity levels normal, reduced and none. The highest value of one of the three parameters determines the patient’s resilience capacity level (RCL). For therapeutic capability an RCL 1 (normal) means that all therapy options can be performed, while RCL 4 (none) means that therapy options are extremely limited or non-existent due to cognitive and/or physical frailty of the patient. Oral hygiene ability RCL depends mostly on the question whether the patient has the ability to perform oral and prosthetic hygiene independently or whether a third person partially or completely performs the oral and prosthetic hygiene of the patient. Self-responsibility describes if the patient acts on his/her own responsibility, independently and autonomously.

**Table 1:** Classification of the Oral functional capacity with resilience capacity level (RCL) and the three parameters therapeutic capability, oral hygiene ability and self-responsibility

Resilience capacity level (RCL)	Therapeutic capability	Oral hygiene ability	Self-responsibility
RCL 1 Normal	Normal	Normal	Normal
RCL 2 Slightly reduced	Slightly reduced	Slightly reduced	Normal
RCL 3 Greatly reduced	Greatly reduced	Greatly reduced	Reduced
RCL 4 No resilience	None	None	None

The evaluation itself is independent of factors such as age, dental status and financial situation. It takes into account a variety of aspects influencing the feasibility of providing treatment to a patient. Some typical aspects to be considered when assessing therapeutic capability include but are not limited to the risk of general medical incidents, drug interactions, transportability, and limitations of patient positioning on the chair, feasibility of diagnostic procedure, manual dexterity and ability to open the mouth for longer periods. Within the scope of oral hygiene ability factors such as visual acuity, handgrip strength, need of help with oral hygiene are assessed. Self-responsibility includes aspects like visiting behaviour/dental service uptake, expression of will, who is the responsible person for decisions, etc. [34] The scale for each parameter of the OFC as well as the instrument itself is well established in this specialized clinic. It is currently undergoing validation and preparation for publication.

The DMF/T index (D - decayed, M - missing, F - filled, T – teeth; related to 28 teeth) is a measure of caries experience. It was used with the knowledge that it is impossible to determine whether a tooth was lost due to caries or other reasons (e.g. trauma, periodontal disease) [35].

Failure rates mentioned in the evaluation refer to biological complications on teeth previously treated under GA. These include secondary caries, fractures (spontaneous or due to trauma) as well as apical translucency.

In Switzerland all dental treatment is financed by the patient. In case the patient is unable to work due to a disability or has an insufficient income, government agencies (Disability Insurance, supplementary benefits to pension) verify eligibility for support. Treatment has to comply with Swiss regulations for social medicine and limitations in terms of permissible treatment (e.g. limited approval for endodontics and fixed prosthesis) [36].

The costs for the anaesthetic services required for a dental treatment session under GA for people with disabilities are generally covered by the health insurance. Only in rare cases are dental treatment costs covered by the accident or health insurance for example in case of treatment after accidents or treatment due to defined oral/medical conditions.

Every dentist in Switzerland may provide dental treatment for people with disabilities and apply for funding. However, limitations frequently exist in terms of spatial and technical requirements as well as personnel required for domiciliary or in-office treatment of people with disabilities.

An anaesthetic fitness assessment was carried out as part of the pre-GA assessment by an experienced anaesthesiologist. The aneasthesiologist determined fitness for anaethesia in close co-operation with the patient’s GP who provided a comprehensive medical history which may include ECG, cardiac echography, spirometry, laboratory parameters. The patient has to be in good general health and sufficiently fit to walk up to the first floor without limitations. Physical fitness can be difficult to assess in people with disabilities which highlights the significance of the clinical examination. Out-patient GA is only provided for patients with ASA classes I to III [37]. Conditions excluded in the given setting include syndromes which make conventional intubation impossible (e.g. Pierre Robin Syndrome, Goldenhar Syndrome etc.) and patients who, due to their medical conditions, require intensive and extended monitoring during the recovery period (e.g. Myasthenia gravis, Chorea Huntington).

For five exceptionally complex cases included in the analysis inpatient treatment had been arranged in cooperation with the clinic for oral and maxillo-facial surgery.

After GA treatment patients are recruited into a recall system with a recall interval of 12 months augmented by 2–4 oral hygiene sessions.

The statistical evaluation was descriptive using SPSS Version 23 [38].

All data were collected from patients who gave their informed consent themselves or through legal representatives. The study was approved by the data protection officer of the Canton of Zurich and classified and approved by the Ethics Committee of Zurich as not requiring approval (ID: Req-2018-00597).

## Results

### Study population

On average, the clinic performs about 5000 treatment sessions for 3000 patients per year. There were a total of 456 appointments for a GA pre-assessment (people with disabilities, people with dementia, people with addiction and psychiatric diseases, people with dental phobia) within 10 years. For this assessment a dentist was available once a week.

Out of these 456 GA pre-assessment appointments for the various vulnerable patient groups listed above, a total of 233 patients with disabilities were registered for an anaesthesia appointment during the observation period of 10 years. Out of these, 12 patients were excluded from the analysis because they were under 18 years of age. This resulted in a total of 221 patients who attended the GA pre-assessment which were included in the analysis (age at the time of the GA pre-assessment median: 37.8 years, range 18–83 years; male *n* = 128, 57.9%). Other vulnerable patients mentioned above (people with dementia, people with addiction and psychiatric diseases, people with dental phobia) were not included in the following analysis. Patients could be tracked post-operatively over a mean period of 3.0 years (± 2.8 years) (Table 2).

**Table 2:** Overview of specific parameters

**a) Patient-specific parameters [*n*** **= 221]**		
Age at the time of appointment for general anaesthesia pre-assessment [years]	Mean	37.8
	SD +/−	15.9
	Median	36.0
	Range	18–83
Sex [n / %]	male	128 / 57.9
	female	93 / 42.1
Observation period [years]	Mean	3.0
	SD +/−	2.8
	Median	2.1
	Range	0–9.8
**b) Number of GA and pre-assessments**		
Number of appointments for general anaesthesia pre-assessment [n]		221
Main subjective reason for application [n / %]	(Suspicion of) Pain	80 / 36.2
	Dental examination	95 / 43.0
	Referral from medical practitioner	1 / 0.5
	Desire for rehabilitation	7 / 3.2
	Referral by dentists	38 / 17.1
Decision for a GA [n / % in relation to all appointments for GA pre-assessment (*n* = 221)]		154 / 69.7
Total number of GA (more than one GA possible per patient over the observation period) [n]		205
Number of GA per patient [n / % in relation to the number of patients receiving a GA (*n* = 154)]	One	113 / 73.4
	Two	33 / 21.4
	Three or more	8 / 5.2
Objective dental indication (GA 1) [n / %]	(Suspicion of) Pain	97 / 63.0
	Dental examination	47 / 30.5
	Referral from medical practitioner	2 / 1.3
	Desire for oral rehabilitation	1 / 0.7
	Referral from other dentist	7 / 4.5
**c) Number and type of teeth treated as well as failures and their further therapy**		
Number of teeth in need of treatment [n / %]		850 / 100
Number of teeth in need of a conservative treatment [n]	As proportion of all teeth in need of treatment (*n* = 850)	442 / 52.0
	of which: - composite - glass ionomer cement - amalgam	394 / 89.1 41 / 9.3 7 / 1.6
Tooth extractions (as proportion of all teeth in need of treatment (*n* = 850)) [n / %]		389 / 45.8
Endodontic treatment (as proportion of all teeth in need of treatment (*n* = 850)) [n / %]		19 / 2.2
Failures		
In relation to: [n / %]	- Number of teeth in need of treatment	43 / 5.1
	- Number of teeth in need of a conservative treatment	43 / 9.7
Type of failure [n / %] (related to number of failures (*n* = 43))		
	Secondary caries	40 / 93.0
	Fracture	3 / 7.0
	Apical translucency	0 / 0
Further therapy of teeth affected by failure [n / %]		
	Extraction	10 / 23.3
	Conservative therapy	33 / 76.7

^a^ Patient-specific parameters of all patients with disabilities who were registered for general anaesthesia pre-assessment; ^b^ Number of general anaesthesia pre-assessments and general anaesthesia performed; ^c^ Number and type of teeth treated as well as failures and their further therapy [n/% and n] (*n* = 221)

**Fig. 1:**
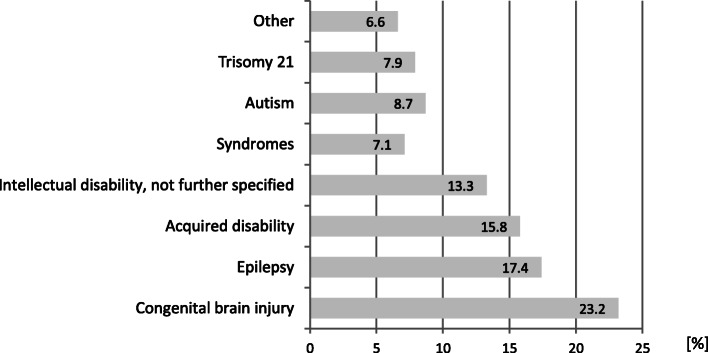
Diagnoses among people with disabilities (period: 2007–2017) [%]. (*n* = 241 diagnoses in 221 patients who underwent a GA pre-assessment, multiple answers were possible)

The disabilities (*n* = 241, multiple responses of diseases were possible) were mostly congenital (*n* = 56, 23.2%) (Fig. 1).

**Fig. 2:**
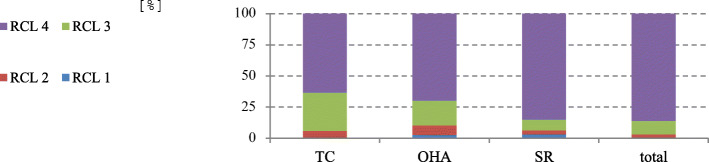
Oral functional capacity (TC - therapeutic capability; OHA - oral hygiene ability; SR – self-responsibility; RCL - resilience capacity level) (*n* = 221) of all patients who underwent a GA pre-assessment independent of individual medical diagnosis of disability

Oral functional capacity (Table 1) shows that people with disabilities had low resilience capacity (over 80% were not self-reliant) (Fig. 2).

### General anaesthesia pre-assessment

Of all patients with disabilities (*n* = 221) who attended the GA pre-assessment and were included in the analysis, 69.7% (*n* = 154) received dental treatment under GA based on the findings and indications listed in Table 2. Sixty-seven (30.3%) of the 221 patients did not receive GA after the first GA pre-assessment (no treatment necessary *n* = 38; treatment at another location *n* = 12; treatment necessary but did not return after pre-assessment *n* = 14; deceased *n* = 2; moved *n* = 1).

Most patients or their relatives/caregivers/legal guardian stated the desire for a dental examination or pain or the suspicion of pain as the main reason for an application for dental treatment under GA (Table 2).

**Fig. 3:**
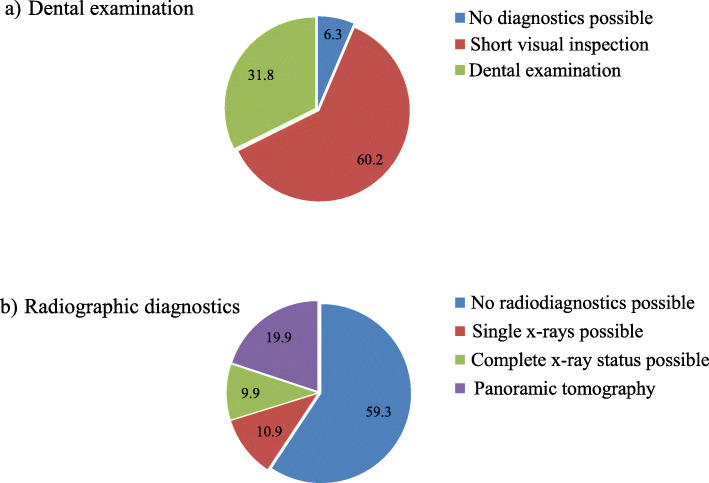
Diagnostic procedures performed at the time of GA pre-assessment in all patients (*n* = 221) [%]. **a** Extent of dental examination. **b** Extent of radiographic diagnostics

Diagnostics within the scope of the GA pre-assessment were limited. In almost two-thirds of the patients, only visual findings and no X-ray diagnosis were possible (Fig. 3).

### General anaesthesia

For *n* = 153 cases data were available on waiting times between GA pre-assessment and first dental treatment under GA. Mean waiting times were 32 weeks (±45.5 weeks) (median: 21.0 weeks, range 0–322 weeks). Out of the 154 patients for whom a decision for a treatment under GA was made in the GA pre-assessment, 73.4% patients (*n* = 113) received one, 21.4% (*n* = 33) received two, and 5.2% (*n* = 8) received three treatment sessions under GA during the observation period. A total of 205 dental treatment sessions were performed under GA in these 154 patients (Table 2). The GA were predominantly outpatient (*n* = 200; 97.6%) and five (2.4%) required admittance to hospital.

The dentist almost twice as often suspected pain on the basis of the dental evaluation as the relatives/carers/legal guardians and therefore recommended GA treatment (Table 2).

Median duration of the first GA was 180 min (range 60–420 min). The duration of the GA was shorter with increasing numbers of GA (2nd GA: median 150 min, range 50–360 min; 3rd GA: median 150 min; range 120–210 min).

The mean interval between the first and second anaesthesia was 3.5 years (SD ± 2.1 years), between the second and third anaesthesia 3.3 years (SD ± 2.8 years).

### Dental findings and procedures

**Fig. 4:**
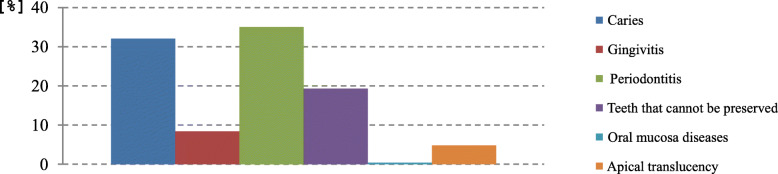
Diagnostic findings at the time of the first general anaesthesia in percent of number of teeth in need of treatment (*n* = 850) [%]. (*n* = 249, multiple answers were possible)

At the first GA, single-tooth X-rays were taken if this had not been possible at the time of the GA pre-assessment, and a full dental assessment as well as preventive dental care (professional oral hygiene treatment) was performed covering hard and soft tissues. Diagnostic results are presented in Fig. 4. Apical translucency of teeth and oral mucosa diseases were rare (Fig. 4).

Dental treatment, in addition to diagnostic imaging (x-ray) and professional tooth cleaning, concentrated mainly on conservative (*n* = 442, 52%) and surgical (*n* = 389, 45.8%) procedures, which were almost evenly distributed. Endodontic treatment (*n* = 19, 2.2%) was rare. (Table 2, related to total number of teeth in need of treatment *n* = 850) The extraction of all remaining teeth under GA in the upper jaw alone was only performed for two patients (1.3%). All remaining teeth in the upper and lower jaw were removed simultaneously in three patients (1.9%). The molars in the maxilla and mandible were most frequently affected by extractions or conservative treatment. Endodontic treatment was predominantly performed in the maxillary and mandibular anterior regions.

The failure rate, related to the number of all teeth in need of treatment (*n* = 850) irrespective of the treatment provided, was 5.1% (*n* = 43). Out of these, the failure rate related to all teeth treated conservatively (*n* = 442) was 9.7% (*n* = 43). In most cases, the failure of a treatment was due to secondary caries (*n* = 40, 93.0%). The teeth affected by failure were subsequently treated conservatively (*n* = 33, 76.7%) or extracted (*n* = 10, 23.3%) (Table 2).

### Additional evaluations

#### DMF/t

**Fig. 5:**
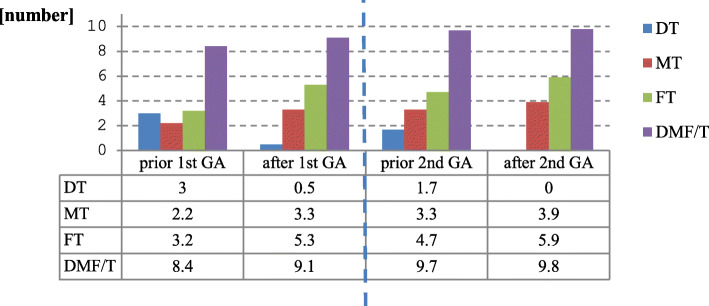
Comparison of DMF/T Index before and after dental treatment in general anaesthesia (GA) for the first and second GA performed (*n* = 34 †). †Complete data records available, in patients who had received two general anaesthesia sessions. (DT – decayed teeth; MT – missing teeth; FT – filled teeth)

The DMF/T index rose slightly over time. The number of decayed teeth before GA treatment decreased over time as the number of filled and missing teeth increased (Fig. 5).

#### Indications

The indications for performing a GA changed in all subjects over time. The indication for the first GA (*n* = 154) was mainly based on suspected pain (*n* = 97, 63%). The main indication for subsequent GA during the observation period were for dental assessment and examination (before 1st GA (*n* = 154): *n* = 47, 30.5%; before 2nd GA (*n* = 33 ‡): *n* = 18, 45%), as well as suspected pain (before 2nd GA (*n* = 33 ‡): *n* = 19, 47.5). (‡ number of patients with two or more GA).

#### Dental emergencies

In patients for whom GA treatment would have been necessary from a dentist’s point of view, but who did not receive it for health reasons or due to the wishes of relatives/carers, no dental emergencies (abscesses, pain, etc.) occurred despite the confirmed treatment need. From this group, only patients without medical contra-indications and a sufficient level of compliance received preventive treatment. (Please see section “Dental recalls”).

Dental emergencies in patients previously treated with GA were very rare. Over the individual observation period per patient, only two dental emergencies (fractures of teeth) concerning teeth previously treated under GA (0.2% (based on the number of all treated teeth *n* = 850)) were observed in all patients treated under GA.

#### Dental recalls

Of all patients analysed (*n* = 221) 67 (30.3%) did not receive a GA or dental recall at the clinic after the first GA pre-assessment because no treatment was necessary (*n* = 38), treatment was provided at another location (*n* = 12), treatment was necessary but the patient did not return after pre-assessment (*n* = 14), and patients died (*n* = 2) or moved (*n* = 1).

Of all patients receiving at least one GA (*n* = 154) 129 patients (83.8%) received the first treatment under GA soon after the GA pre-assessment without any dental recalls between GA pre-assessment and GA treatment session.

Out of these 129 patients, six (4.7%) could not receive any further chairside appointment due to greatly reduced compliance (extraneous aggression, auto-aggression under stress, etc.). Since the diagnosis in these severe cases is based exclusively on external anamnestic observations (no visual findings possible, no further appointment in the clinic) the relatives and the nursing staff were instructed in oral hygiene and the recognition of possible symptoms of potential oral pain. In addition, depending on the possibility of having the oral hygiene performed by third parties, a future periodic visit was made directly in GA (indication: routine examination) without any further pre-assessment chairside.

Twenty-five (16.2%) of the 154 patients who received at least one treatment under GA, had joined the recall system of the clinic before they received their first treatment under GA (number of recalls before the first treatment under GA: median 3, range 1–27). Among these patients (*n* = 25), 23 patients had been seen by a dentist for a routine recall and prophylaxis while two patients (8%) had received dental prophylaxis from a dental hygienist only before the first treatment under GA. For these two patients, the decision for a treatment under GA was made during the oral hygiene session together with the patient and the accompanying relatives/carers/legal representatives as well as a dentist who was consulted by the dental hygienist.

Following the first GA, 106 patients out of the 154 who received a first GA, entered the recall system of the clinic and were seen by a dentist for their following annual recall sessions. Out of these 106 patients, 24 patients for whom at least a chairside professional dental prophylaxis was possible attended a yearly dental recall with a dentist in the clinic when a decision was made together with the relatives/caregivers/legal guardian as to whether further treatment in GA was necessary or whether dental care within the framework of dental hygiene and recalls was sufficient. One of the following criteria was seen as indication for further GA treatment: a) status after trauma, b) acute clinical findings (e.g. swelling, chipped teeth with exposed pulp), c) changes in character or behaviour that were not due to any other cause (after examination by medical practitioner) (e.g. pronounced restlessness, aggression etc. as suspicion of pain), d) no possibility for third parties (dental staff or relatives/caregivers/ for the people with disabilities) to carry out oral hygiene in combination with considerable inflammation or halitosis, e) desire for dental examination/treatment by third parties (doctors, other dentists on referral).

## Discussion

In the present analysis, data on dental care and treatment of people with disabilities over a period of 10 years were evaluated. The data provide an overview of dental care needs and the additional evaluations made (change in DMF/T, change in indications for a treatment under GA in the following GA pre-assessments, occurrence of emergencies etc.) as well as preferences in dental therapy options in patients who require dental treatment under GA due to a disability.

### Limitations

Data analysis was based on retrospectively extracted data from patient files. The authors have to assume that in line with legal requirements diagnostic and therapeutic procedures have been accurately entered into the patient files. Data are specific for conditions in Switzerland and cannot necessarily be generalised to other countries as choice of treatment is partly determined by Swiss regulations governing public funding [36]. For example extractions are favoured over endodontics for financial reasons. The rehabilitation of chewing function has to be achieved primarily with simple, economic and expedient measures [36] favouring removable rather than fixed prosthodontics. Fixed prosthodontics will only be funded in justified cases where there often is no alternative. The regulations are primarily geared at Swiss citizens on a low income and available benefits follow economic aspects. Medical supply of the general Swiss public on a low income is thus secured. However, the regulations make no allowance for the specific needs and requirements of people with disabilities. Not all medically possible therapeutic options are supported and funded. This aspect particularly affects people with disabilities as there is little leeway to adapt treatments plans to their individual situation and specific needs. Only for patients receiving financial support, for example from their families, is it possible for the dental team to implement treatment options that fully embrace the principles of inclusion, equality and ultimately justice.

The ethical principles of non-maleficience and beneficence can be even more difficult to implement [28]. Limitations due to the patient’s condition including behavioural aspects can severely limit provision of regular standard oral hygiene measures even by carers. Such limitations, which may also apply to the provision of professional prophylaxis, effectively limit the implementation of the principle of beneficience and ultimately foster oral disease. The principle of justice ought to be guiding all decisions taken on the meso-level of health insurances and third-party financing institutions and on the makro level of law- and health policy makers on the financing of preventive oral health measures and care for people with disabilities. Currently all too often health care providers even in wealthy jurisdictions are severely restricted in their treatment choices by regulations and policies that seem to be designed to primarily limit costs rather than to facilitate and support health.

Access to dental care for people with disabilities who are dependent on carers who could be nurses, social worker, nursing assistants, supervisors for the people with disabilities, family members etc. generally depends on the importance the carers attach to oral health of the dependent person. Barriers for caregivers, who were mostly professional nursing staff, described in the literature are a lack of time associated with workload, and poor knowledge of dental diseases and their causes [39]. We can only speculate, that the lack of oral-health knowledge in social workers, family members and other carers without formal training in nursing, may be significantly larger.

The present analysis is retrospective reflecting treatment concepts under GA current in Switzerland at the time of treatment. Dental treatment under GA often appears to be different from usual dental treatment. The literature indicates a tendency towards more extractions instead of tooth preservation (e.g. endodontics) to avoid possible failures and complications [40]. However, sometimes a large number of restorative procedures are performed under GA [9, 41]. Conservative treatment and extractions carried out under GA in Zurich were almost evenly distributed. Endodontic treatment was rare because it is particularly time consuming and may carry an increased risk of long-term complications [18]. The literature questions the use of composite filling materials for patients with impaired oral hygiene and/or limited professional aftercare depending on material and location in the oral cavity due to increased failure rates (e.g. failure rates for composite restorations Tate et al. 30%; Molina et al. 15.5%) [22, 42]. The present analysis has a slightly lower failure rate of conservative treatment of 9.5% over the observation period. It could be speculated, that the mean oral hygiene of the participants in the current study was better than in other studies dealing with composite fillings in patients with impaired oral hygiene due to an established recall for preventive measures applied by oral hygienists wherever possible. The present analysis has shown a similar failure rate of conservative restorations in people with disabilities treated under GA as reported by Alvanforoush et al. for a 10-year period for posterior restorations in patients treated without GA (Alvanforoush et al. failure rate: 13.3%) [43].

People with disabilities are frequently not able to perform sufficient oral hygiene on their own and are therefore dependent on the support of their carers. Even the abilities of dental professionals can be limited by patients’ inability to tolerate procedures, lack of compliance, non-cooperation and defensive or aggressive behaviour. It is thus not surprising to find that the quality of individual routine daily oral hygiene provided at home varies greatly in people with disabilities who require GA treatment. The authors wish to highlight the importance of any improvements achievable in daily routine oral hygiene measures by relatives and caregivers for the long-term preservation of treatment success and the reduction of disease burden and future treatment needs. Caregivers accompanying people with disabilities to the clinic are routinely given instruction and motivation regarding oral hygiene and denture care during recall sessions by dentists and dental hygienists. However, there are limitations on time during these sessions not least because of the limited tolerance of patients for extended sessions. As highlighted above, many caregivers, attendants and accompanying social workers have no medical background and would thus require specially tailored and more extensive training than nursing staff. This training is currently lacking. Efforts by the dental profession, third-party funders, and health policy makers to develop and implement programs to improve oral hygiene skills of all caregivers could reduce the treatment need and associated risks for the patients as well as the financial burden for funders and secure the long-term success of complex GA treatment.

Currently there is a trend towards a more conservative approach during treatment of people with disabilities under GA to avoid high numbers of dental extractions [29]. Equity and justice demand that treatment plans for patients with disabilities treated under GA are developed observing the same principles applied in developing treatment plans for other patients with the same aims of tooth preservations, avoidance of dental extractions and similar therapeutic outcomes [29]. The success described in the literature of pulpotomies in permanent teeth, both, with reversible and irreversible pulpitis, invites consideration of this option for treatment under GA as well [44]. The literature favours complete pulpotomies under GA for the vital preservation of deeply destroyed carious teeth as a timesaving method avoiding the risks and effort associated with endodontic treatment [29]. Even root canal treatment under GA [40, 41, 45–48], in spite of the time needed and the potential risks and complications, is increasingly reported in the literature.

New options in prosthetic therapy have opened up. Pre-formed stainless steel crowns have been proposed as permanent restorations placed under GA on permanent teeth [49].

The use of CAD/CAM (Computer-Aided Design and Manufacturing) manufactured ceramic restorations is being discussed [50]. Further research and long-term studies are required to establish the success rates of these techniques before they could be recommended as routine treatment under GA. For some patients with severe cognitive impairment who are unable to maintain oral hygiene themselves the literature presents a treatment option with a positive outlook using dental implants placed under GA [51, 52]. It is stressed that the long-term success largely depends on patient selection taking into account possible complications and their management [52]. The authors are very cautious in their indication for implants highlighting the importance of a regular follow-up including professional oral hygiene care.

Special needs patients often have a reduced ability to perform oral hygiene themselves and access to dental services is reduced. This in turn can result in an increased risk of caries and periodontal disease [5–7]. This hypothesis is clearly reflected in the numbers of carious or unsustainable teeth in this analysis.

A comparison of the present analysis with other sources [53–58] is difficult due to the heterogeneity of the investigated populations (Table 3). The DMF/T value of the examined patients is difficult to compare due to a broad age range. Based on the mean age (36.7 years) of all test persons in this analysis to classify the DMF/T value, the following statements can be made: People with disabilities have a significantly lower DMF/T value in the present analysis compared to other studies [53–58] (people with disabilities in the present analysis DMF/T 7.9 (prior 1st GA) - 9.4 (prior 2nd GA)) (Fig. 5). This can be explained by the fact that although the number of DT (DT before treatment in GA: 3 (prior 1st GA) or 1.7 (prior 2nd GA)) (Fig. 5) was higher than in other studies [53–55], the FT here was significantly lower due to the lower number of dental contacts (treatment only possible in GA) (FT before treatment in GA: 3.1 (prior 1st GA) or 4.6 (prior 2nd GA) (Fig. 5); FT in DMS V: 8.6) (Table 3). The cohort analysed here therefore appears to be healthier in terms of dental status (lower DMF/T values) than comparable groups of people with disabilities of the same age (DMF/T values shown in Table 3) [53–58].

**Table 3:** Comparative consideration of studies with similar populations/age limits with regard to the DMF/T value

	Current analysis (Mean 37.8 years)	DMS V [53] *n* = 966 (35–44 year olds)	DMS V [54] (65–74 year olds)		Schulte et al. (2011) [55] *n* = 420 (median age 30.8 years ±10.2 years)				Schulte et al. (2013) [56] *n* = 428 (median age 35,5 years ±11.0 years)					Cichon und Donay(2004) [57] *n* = 745 (total in all age groups) (mentally disabled 41.2%, physically disabled 16.5%, mentally-physically disabled 42.3%)		Schnorrenberg (2010) [58] *n* = 1100 (mentally disabled, physically disabled, mentally-physically disabled)			
			younger seniors (total) *n* = 1042	severely disabled younger seniors *n* = 199	age [years]				age [years]					age [years]		age [years]			
					18–24	25–34	35–44	45–70	18–24	25–34	35–44	45–54	55–64	35–44 (*n* = 128)	≥45 (*n* = 66)	25–34	35–44	45–54	> 54
**DT**	1.6–3.5	0.5	0.5	0.9	1.01	1.11	0.86	0.82	1.69	2.15	1.82	2.24	2.45	4.3	3.3	3.0	3.3	2.9	3.6
**MT**	2.1–4.1	2.1	11.4	14.5	0.51	1.81	4.15	11.75	1.44	3.00	6.11	11.51	13.32	6.9	11.1	4.8	6.8	10.6	18.5
**FT**	2.5–5.6	8.6	6.1	4.5	2.88	5.96	8.44	6.79	3.64	4.92	5.73	4.03	2.68	5.0	4.2	3.9	4.3	4.0	4.2
**DMF/T**	8.1–9.3	11.2	17.7	19.9	4.39	8.88	13.45	19.36	6.78	10.09	13.66	17.77	18.45	16.2	18.6	11.7	14.4	17.5	21.4

A further study on the assessment of the oral health status of athletes with intellectual disabilities (mean: 27 years) at the Special Olympics (2008–2016) resulted in lower DMF/T values (DMF/T 7.6 (2008), 7.3 (2010), 7.1 (2012), 6.7 (2014) and 5.6 (2016)) compared to the present analysis with a DMF/T of 7.9–9.4 [59]. A possible explanation could be that the majority of the athletes examined (95%) stated that they could carry out their oral hygiene independently [59]. It can therefore be assumed that these athletes have a higher degree of independence and have to live with fewer restrictions due to the structured sporting activities. This will also be associated with greater use of dental services. Despite the disabilities, this group of patients often can be treated in the dental chair.

The study by Cichon and Donay (2004) [57] with a comparable cohort (study participants were recruited from the patient clientele of a specialised clinic, including only people with physical or intellectual disabilities), recorded higher DMF/T (cf. Table 3) values than the data presented herein. The reason for this could be that some of the subjects in the present analysis were long-term patients of the clinic already at the anaesthetic clarification date. These patients may have benefitted for an extended period from participation in a in a closely monitored preventive and curative care concept, individualised according to their previous illnesses.

For people with disabilities who are not participating in a professionally organised preventive and curative oral health programme it is imperative that caregivers/legal guardian and relatives are trained to recognise dental problems among those entrusted to them and then to organise an adequate response. A reliable cooperation between caregivers, dentists and anaesthetists would be beneficial with continuously open channels of communication and fast response to any requests for support. It must also be clarified together whether a periodic chairside examination is sufficient to maintain oral health and prevent dental emergencies. Regular dental recalls after GA are essential to re-inforce instructions on oral hygiene [60] and to detect changes. Berkowitz et al. reported that the failure to attend follow-up appointments and the disability itself are potential causes for repeat GA treatment [25]. People with disabilities not participating in regular dental recalls were four times more likely to receive repeat GA treatment than those who attended follow-up appointments regularly. It can therefore be assumed that a regular recall reduces the degree of severity and the number of repeat GA dental procedures [61]. Individual follow-up appointments as well as additional appointments, e.g. for dental cleaning, etc., should be discussed with the caregivers [60]. Furthermore, intensive training on oral hygiene and nutrition [60] at home should be provided and supported by the use of high fluoride toothpastes and sugar-free foods, for example. Dentists and oral hygienists play an important role in oral health education for all of their patients. They should be encouraged to offer specific advice on oral hygiene, nutrition and the importance of regular prophylaxis to people with disabilities and their carers to support inclusion of persons with disabilities. Long recall intervals of 12 months can only be achieved with very good communication and information transfer between the persons responsible for the (dental) medical, nursing and socio-educational care of vulnerable patients. In the literature shorter follow-up intervals of 4–6 months [25] or even only 2 months are suggested [62]. Such short intervals could not be implemented in the specialised clinic due to limited clinic staff and the high demand on carers of the disabled. The recall system of the clinic is well organised. A recall interval of 12 months (dentist) was augmented by 2–4 oral hygiene sessions per year for all patients for whom at least a chairside professional dental prophylaxis was possible. From the 221 patients attending the GA pre-assessment 154 patients either received treatment under GA soon after the GA pre-assessment or entered the recall system of the clinic. Patients who received treatment under GA soon after the pre-assessment entered the recall system afterwards. None of the patients (*n* = 154) was lost to follow-up or missed a recall until the next treatment under GA took place. Six patients could not receive any further chairside appointment due to greatly reduced compliance. They received treatment under GA at regular intervals.

Waiting times of more than half a year (mean 32 ± 45.5 weeks) between GA pre-assessment and treatment under GA were high compared to waiting times for out-patient chairside appointments. They were partly due to limited staff and operating rooms, but also due to time taken for administrative procedures for application and approval of funding from government agencies and health insurances in parallel. Patients` appointments could only be arranged after approval of financial arrangements from third-party funders had been received. There is no data indicating a possible deterioration in oral health due to extended waiting periods.

In the absence of any symptoms and complaints, it is difficult to justify the use of GA solely for the purpose of a routine dental examination. Even if the application of GA in such cases is accepted, it is not clear how long the interval between GA treatment should be in patients who are otherwise uncooperative [63]. There is also no literature on the safety of repeated GA applications for people with disabilities [19]. People with congenital disabilities also reach old age and increasingly suffer from geriatric diseases. The occurrence of combinations of congenital disabilities and geriatric diseases (e.g. trisomy 21 and dementia [63]) will be observed more frequently in the future. The additional geriatric diseases usually increase the risk of GA. The repeated application of GA must therefore be re-evaluated under this aspect. Potential post-operative cognitive dysfunction (POCD) which is assumed to influence quality of life and may increase mortality, is multi-factorial and just one aspect that needs further consideration in this context [64, 65].

In the present analysis, no patient presented with a typical dental emergency such as a dental abscess neither during the pre-assessments nor during the control appointments. With close cooperation between the dentist and carers, early signs of possible dental problems (pain, refusal to eat, restlessness, etc.) in patients can be addressed and, if necessary, treatment under GA can be planned. However, such an approach is only feasible if treatment under GA can be organised promptly if necessary.

In the opinion of the authors, it is therefore important that prophylactic treatment under GA is not performed as a standard procedure, which only serves the purpose of a routine dental examination. A risk-benefit assessment should always be performed and peri-operative complications, which occur more often with increasing age, should be taken into account.

## Conclusion

Dental treatment need is high for people with disabilities requiring treatment under GA. The main indication for treatment under GA is suspected or confirmed pain or dental complaints. Before each decision to perform treatment under GA, the dentist, as the case-manager together with the legal guardian and anaesthetist, have to perform a cost-benefit analysis. Dentists find their contribution to inclusion impeded due to frequently limited financial resources available to people with disabilities and the limitations imposed on therapeutic options by third party funders.

Dental care can be successful, for the benefit of patients with special needs, if all carers work together. The caregivers have to be trained in nutrition control limiting the intake of cariogenic and erosive food, as well as in oral hygiene. The cooperation of caregivers and the dental team helps to avoid dental emergencies in patients who are difficult or very complex to treat. The basis is interdisciplinary knowledge, and financial and personnel resources that enable cooperation between dentists, anaesthetists, nursing staff and relatives.

## Data Availability

The datasets generated and/or analysed during the current study are not publicly available due the ethics approval but are available from the corresponding author on reasonable request.

## Abbreviations

GA: General anaesthesia
D - decayed, M - missing, F - filled, T - teeth: DMF/T index
OFC: Oral functional capacity
RCL: Resilience capacity level
TC: Therapeutic capability
OHA: Oral hygiene ability
SR: Self-responsibility
